# A Human-Derived Monoclonal Antibody Targeting Extracellular Connexin Domain Selectively Modulates Hemichannel Function

**DOI:** 10.3389/fphys.2019.00392

**Published:** 2019-06-11

**Authors:** Gaia Ziraldo, Damiano Buratto, Yuanyuan Kuang, Liang Xu, Andrea Carrer, Chiara Nardin, Francesco Chiani, Anna Maria Salvatore, Gaetano Paludetti, Richard A. Lerner, Guang Yang, Francesco Zonta, Fabio Mammano

**Affiliations:** ^1^CNR Institute of Cell Biology and Neurobiology, Monterotondo, Italy; ^2^Institute of Otolaryngology, Università Cattolica del Sacro Cuore, Rome, Italy; ^3^Shanghai Institute for Advanced Immunochemical Studies, ShanghaiTech University, Shanghai, China; ^4^Department of Physics and Astronomy “G. Galilei”, University of Padova, Padua, Italy; ^5^Fondazione Policlinico Universitario A. Gemelli IRCCS, Rome, Italy

**Keywords:** connexin hemichannels, rare diseases, phage display libraries, therapeutic monoclonal antibodies, molecular dynamics, patch clamp

## Abstract

Connexin hemichannels, which are plasma membrane hexameric channels (connexons) composed of connexin protein protomers, have been implicated in a host of physiological processes and pathological conditions. A number of single point pathological mutations impart a “leaky” character to the affected hemichannels, i.e., make them more active or hyperactive, suggesting that normal physiological condition could be recovered using selective hemichannel inhibitors. Recently, a human-derived monoclonal antibody named abEC1.1 has been shown to inhibit both wild type and hyperactive hemichannels composed of human (h) connexin 26 (hCx26) subunits. The aims of this work were (1) to characterize further the ability of abEC1.1 to selectively modulate connexin hemichannel function and (2) to assess its *in vitro* stability in view of future translational applications. *In silico* analysis of abEC1.1 interaction with the hCx26 hemichannel identified critically important extracellular domain amino acids that are conserved in connexin 30 (hCx30) and connexin 32 (hCx32). Patch clamp experiments performed in HeLa DH cells confirmed the inhibition efficiency of abEC1.1 was comparable for hCx26, hCx30 and hCx32 hemichannels. Of note, even a single amino acid difference in the putative binding region reduced drastically the inhibitory effects of the antibody on all the other tested hemichannels, namely hCx30.2/31.3, hCx30.3, hCx31, hCx31.1, hCx37, hCx43 and hCx45. Plasma membrane channels composed of pannexin 1 were not affected by abEC1.1. Finally, size exclusion chromatography assays showed the antibody does not aggregate appreciably *in vitro*. Altogether, these results indicate abEC1.1 is a promising tool for further translational studies.

## Introduction

Connexins are a family of integral transmembrane proteins (indicated by Cx followed by their molecular weight in kDa, e.g., hCx26 for human connexin 26, encoded by the *GJB2* gene) which form hexameric plasma membrane structures known as “connexons.” A connexon may function as a regular plasma membrane channel, termed “hemichannel,” or dock head-to head with another connexon from an opposing cell and self-assemble into a gap junction intercellular channel (Mammano, 2018).

Partial high-resolution crystal structures have been determined only for hCx26 (Maeda et al., 2009) and sheep Cx46/50 (Myers et al., 2018). However, due to the relatively high sequence similarity across the family, all connexin proteins are thought to share a topology similar to that of hCx26 or Cx46/50, which comprise 4 transmembrane helices (TM1-4) connected by 2 extracellular loops (EC1, EC2) and 1 intracellular loop (ICL). An N-terminal helix (NTH) domain folds into the cytoplasmic channel vestibule and is connected to the pore-lining TM1 helix via a short linker. The ICL, connecting TM2–TM3, and the cytoplasmic C-terminal domain (CTD) were not resolved (Maeda et al., 2009; Myers et al., 2018). The CTD, which is considered to be unstructured, is the most diverse domain and its length is different in each connexin isoform. The fairly conserved sequences of EC1 and EC2 suggest the extracellular vestibule of all hemichannels has a relatively rigid three-dimensional (3D) structure. In MD simulations lasting ∼100 ns, it appears to be the stiffest part of the hemichannel (Zonta et al., 2012) due to the presence of six conserved cysteine residues, three in each loop, forming intramolecular disulfide bonds between EC1 and EC2 (Maeda et al., 2009; Myers et al., 2018).

In a hCx26 gap junction channel, the extracellular docking interface of each connexon comprises hydrogen bonding between Asn54 of EC1 and the main-chain amide of Leu56 in the opposite protomer, and a pair of Gln57 in two diagonally opposite protomers (these residues are highly conserved among connexins). Also EC2 contributes to the connexon-connexon interaction with a complex network of hydrogen bonds and salt bridges mediated by Lys168, Asn176, Thr177 and Asp179 in two opposite protomers (Maeda et al., 2009).

Accurate control of undocked hemichannel gating is crucial for cell survival and organism health. Indeed, “leaky” or more active mutant hemichannels result in cell death when expressed in model cells (Abrams et al., 2002; Essenfelder et al., 2004; Liang et al., 2005; Stong et al., 2006; Dobrowolski et al., 2007, 2008; Lee and White, 2009; Sanchez et al., 2010, 2013, 2014; Tong et al., 2011; Yao et al., 2011; Chi et al., 2012; Kozoriz et al., 2013; Mhaske et al., 2013; Ren et al., 2013; Berger et al., 2014; Patel et al., 2014; Sun et al., 2014; Zhu et al., 2014; Wang et al., 2015; Sanchez et al., 2016; Press et al., 2017; Xu et al., 2017; Srinivas et al., 2019); reviewed in Retamal et al. (2015), Laird and Lampe (2018), and Srinivas et al. (2018).

Recently, a human-derived single-chain fragment variable (scFv) fragment constant (Fc) antibody (scFv-Fc) named abEC1.1 (Qu et al., 2017) was shown to inhibit both wild type (wt) and hyperactive pathological hCx26 hemichannels (Xu et al., 2017). The crystal structure of the scFv domain was solved (Protein Data Base accession code 5WYM) and some of the residues that are critical for its binding to the extracellular domain of hCx26 hemichannels were identified.

The goals of the present study were to characterize further the biophysical properties of this antibody, particularly its selectivity for connexin hemichannels, and to assay its *in vitro* stability, which is a pre-requisite for future *in vivo* delivery to animal models of disease.

## Materials and Methods

### Antibody Production

The gene encoding the antibody scFv domain was cloned into a pFUSE-Fc expression vector (Cat. No. pfuse-hg1fc2, Invivogen, Hong Kong) to generate abEC1.1 as a diabody-Fc fusion protein (Wu et al., 2001) which comprises the entire Fc domain of human immunoglobulin G1 (IgG1) (Silverton et al., 1977; Bujak et al., 2014; Frenzel et al., 2016). In addition, the pFUSE-Fc expression vector was modified to generate a variant of the antibody with a murine Fc (abEC1.1m). For antibody production, a FreeStyle^TM^ 293-F cell line (Thermo Fisher Scientific, Cat. No. R79007), maintained in Freestyle 293 Expression Medium (Thermo Fisher Scientific, Cat. No. 12338026), was stably transfected with the abEC1.1 or abEC1.1m expression vector. Expressed antibodies were purified using HiTrap Protein A HP columns (GE Healthcare, Cat. No. 17-0403-03) with the ÄKTApurifier 100 system (GE Healthcare). After purification, the buffer was exchanged to PBS (pH 7.4) and the antibodies were kept in PBS at 4°C.

### Size Exclusion Chromatography (SEC)

To characterize the *in vitro* stability of abEC1.1 and abEC1.1m by SEC (Fekete et al., 2014; Bobaly et al., 2017), eppendorf tubes containing 4.2 mg/mL of antibody per tube were incubated at 4°C, room temperature (∼22°C), 37°C and 42°C for 3, 5, 7 and 16 days. At each time point, 20 μL of solution were loaded into a Nanofilm SEC-250 column (Sepax Technologies, Inc., DW, United States) and processed at flow-rates of 0.5 mL/min using a 1290 Infinity II liquid chromatography system (Agilent Technologies, Santa Clara, CA, United States). Raw data generated by the instrument (optical density, OD, measured at 405 nm vs. retention time) were normalized to the peak of each chromatogram and plotted using OriginPro 2017 software (OriginLab, Northampton, MA, United States).

### Construction of Connexin-Venus Transfection Vectors

The coding regions of wt *homo sapience* connexin genes^1^ were synthesized (by Shanghai Sangon Biological Engineering Technology & Services Co., Ltd.) without stop codon and subcloned into a pcDNA3.1(+) mammalian expression vector (Cat. No. V79020, Thermo Fisher Scientific) that had been previously modified for C-terminal fusion of connexin genes with Venus, a circularly permuted mutant of the yellow fluorescent protein (YFP) (Beltramello et al., 2005). After transformation into *Escherichia coli* (TOP10, Cat. No. C404010, Thermo Fisher Scientific), miniplasmid preparation and restriction enzyme analysis were performed to identify positive clones. To verify that PCR amplification did not introduce unwanted mutations, all constructs were sequenced (by Eurofins Genomics S.r.l., Milan, Italy) using standard primers complementary to the plasmid common regions of the constructs, adjacent to the target open reading frames (ORF). Both orientations were sequenced when sequencing was insufficiently reliable as a consequence of the length of the ORF. The primers sequences used for this purpose were:

T7: 5′-TAATACGACTCATAGGG;

myc-ddk-rev: 5′-TGCCAGATCCTCTTCTGAGATGAG.

### Patch Clamp Electrical Measurements

Communication–incompetent HeLa DH cells (Cat. No. 96112022, Sigma-Aldrich/Merck, Milan, Italy) were seeded onto round glass coverslip (Cat. No. FIS#12-542A, Thermo Fisher Scientific) and maintained in Dulbecco’s modified Eagle’s medium (DMEM, Cat. No. 41965039, Thermo Fisher Scientific) containing 10% (v/v) fetal bovine serum (FBS, Cat. No. 10270-106, Gibco-Invitrogen) and 1% penicillin/streptomycin (Cat. No. 15070-063, Gibco-Invitrogen). Twenty four hours after plating, the Lipofectamine 3000 transfection reagent (Cat. No. L3000-015, Thermo Fisher Scientific) was used to transiently transfect HeLa DH cells at 25-30% confluence with one of the connexin-Venus plasmids.

For patch clamp recordings, a double stage vertical puller (PP-830, Narishige) or horizontal laser-based puller (P-2000, Sutter Instrument) were used to fabricate patch pipettes from glass capillaries (G85150T-4, Harvard Apparatus, Edenbridge, United Kingdom). Pipettes were filled with a potassium aspartate-based intracellular solution (ICS_KAsp_) containing (in mM): 115 KAsp, 10 NaCl, 10 KCl, 1 MgCl_2_, 10 HEPES, 1 CaCl_2_ and 4 BAPTA tetrapotassium salt (pH 7.2, 311 mOsm) and filtered through 0.22-mm pores (Millipore). Twenty four hours after transfection, glass coverslips with adherent cells were transferred to the stage of an upright fluorescence microscope (BX51, Olympus) equipped with differential interference contrast (DIC) optics. Cells were continuously superfused at 2 ml/min at 20–23°C with a sodium chloride-based extracellular solution (ECS_NaCl_) containing a reduced (0.2 mM) Ca^2+^ concentration ([Ca^2+^]_e_) and (in mM): 140 NaCl, 5 KCl, 10 HEPES, 2 sodium pyruvate, 4 tetraethylammonium chloride (TEA-Cl), 1 MgCl_2_, 4 CsCl and 5 glucose (pH 7.4, 323 mOsm). Filled patch pipettes had resistances of 4–6 MΩ when immersed in ECS_NaCl_.

Hemichannel currents were assayed in ECS_NaCl_ while keeping cells near their zero-current potential (between -20 and 0 mV) under whole cell patch clamp recording conditions. Cells were transiently depolarized to +40 mV for 20 s followed by a ramp down -40 or -60 mV and subsequently held at this negative potential for up to 1 min before stepping back to the zero-current potential. The ramp from positive to negative potentials was preferred to a step to minimize stress to the plasma membrane. To estimate plasma membrane leak currents, connexin hemichannels were blocked by adding either CaCl_2_ (2 mM) or ZnCl_2_ (100 μM) to the superfusion medium (Xu et al., 2017).

For antibody application, the opening of a glass micropipette connected to a pneumatic pico-pump (PV820, World Precision Instruments Inc., Sarasota, FL, United States) and filled with the ECS_NaCl_ extracellular solution supplemented with abEC1.1 (952 nM) was positioned near the patched cell. During antibody delivery, the superfusion was stopped.

Negative control experiments were performed in HeLa-Panx1-YFP cells (Yi et al., 2017) grown in DMEM with 10% FBS, containing G418 (Cat. No. 4727878001, Sigma-Aldrich/Merck) at a final concentration of 1 μg/mL at 37°C in an incubator supplemented with 5% CO_2_. Pannexin currents were inhibited using the following compounds, all from Sigma-Aldrich/Merck: p-(Dipropylsulfamoyl)benzoic acid (probenecid, Cat. No. P-8761), 3β-Hydroxy-11-oxoolean-12-en-30-oic acid 3-hemisuccinate (carbenoxolone, CBX, Cat. No. C4790), 4,4-Diisothiocyanatostilbene-2,2-disulfonic acid (DIDS, D3514) and Fast Green FCF (Cat. No. F7252-5G).

### Dye Uptake Assay

HeLa DH cells expressing the connexin of interest were imaged in an upright fluorescence microscope (Bergamo II, Thorlabs Inc., Thorlabs Inc., Newton, NJ, United States) using a 25× water immersion objective (XLPLN25XWMP2, 1.05 N.A., Olympus Corporation, Tokyo, Japan) and a programmable illumination system (pE-4000, CoolLED Ltd., Andover, United Kingdom) coupled to the epifluorescence port of the microscope via 474/24 nm bandpass filter (Semrock, Rochester, NY, United States) and dichroic beamsplitter (410/504/582/669 nm BrightLine quad-edge, Semrock). Fluorescence emission was filtered through a 525/39 nm bandpass filter (Semrock) to form images on the sensor of a scientific CCD camera (1501M-USB-TE, Thorlabs). HeLa DH transfectants were maintained in a divalent-free extracellular medium (ECM) containing (in mM): 140 NaCl, 5 KCl, 10 HEPES, 2 sodium pyruvate, and 5 glucose (pH 7.4). Five non-overlapping fields of view were rapidly imaged under fixed illumination and recording conditions (time *t*_0_) using a precision programmable motorized stage (HLD117NN, Prior Scientific Instruments Ltd., Cambridge, United Kingdom). Thereafter, cells were switched to ECM supplemented with 2 mM Lucifer Yellow (CH dilithium salt, Sigma-Aldrich/Merck, Cat. No. L0259) to promote dye uptake (DeVries and Schwartz, 1992; Saez and Leybaert, 2014). After 30 min of incubation (time *t*_30_), cells were washed three times with ECM supplemented with 2 mM CaCl_2_ (ECM_Ca_) in order to remove dye molecules bound to the outer plasma membrane leaflet and to limit dye escape from cell cytoplasm through open hemichannels. Thereafter, a new set of 5 images were rapidly acquired from the same fields of view. For off-line image processing, regions of interest (ROIs) were drawn over individual cells and ROI average fluorescence intensities were computed. To test antibody efficacy, batches of cells which had been pre-incubated for 30 min in ECM supplemented with abEC1.1 (952 nM) were imaged as explained above. Cells transfected with an empty pcDNA3.1(+) vector (mock transfected) were used as controls for aspecific dye uptake effects. The distributions of fluorescence values, sampled before and after Lucifer Yellow application, were compared statistically using the Mann–Whitney *U* test (Mann and Whitney, 1947).

### Molecular Modeling and Dynamics

The abEC1.1 model was derived by homology from two different templates, one for the scFv domain and the other for the Fc domain, which were interconnected by a linker using the Swiss Model website (Biasini et al., 2014). The scFv domain was then docked to the extracellular domain of a published model of hCx26 hemichannel embedded in the plasma membrane (Zonta et al., 2012) using the ClusPro 2.0 server (Comeau et al., 2004), and the antibody docking mode (Brenke et al., 2012). Among the 50 docking configurations generated by the software, we selected the only one in which the 3 complementarity determining regions (CDRs) of the abEC1.1 heavy chain faced the EC1 loop of hCx26. Configuration stability was tested by performing a MD simulation using the Gromacs 4 package (Pronk et al., 2013) and the Amber03 force field (Duan et al., 2003). Specifically, the atomistic model system, containing the hCx26 hemichannel inserted in a phospholipid bilayer and the docked antibody, underwent a short energy minimization in vacuum and was subsequently solvated with full atom TIP3P water, containing Cl^-^ and K^+^ ions at a concentration of ∼0.15 M in order to mimic a physiological ionic strength. After solvation, the total number of atoms was around 2.53 × 10^5^. We then performed an equilibrium MD simulation under periodic boundary conditions at constant pressure for 150 ns and analyzed the last 10 ns after equilibration. Temperature *T* and pressure *P* were kept constant, at 300 K and 1 atm, respectively, using the Berendsen thermostat and barostat (Berendsen et al., 1984). Fast smooth Particle–Mesh Ewald summation (Darden et al., 1993) was used for long–range electrostatic interactions, with a cut off of 1.0 nm for the direct interactions.

## Results

### *In silico* Analysis of abEC1.1 Interaction With the Extracellular Domain of the hCx26 Hemichannel

**FIGURE 1:**
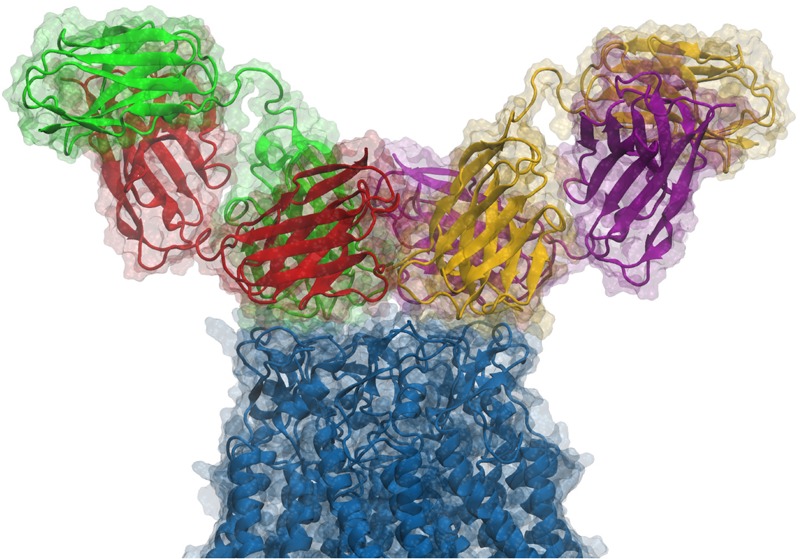
Side view of an antibody pair binding a hCx26 hemichannel. According to our model and to the experimental determination of the stoichiometry of binding, two different antibodies can simultaneously bind the extracellular region of a single connexin hemichannel. Each antibody is a diabody-Fc, i.e., a dimer of two scFv-Fc polypeptides where the heavy chain (HC) and light chain (LC) from one scFv, pair with the complementary domains of a second scFv (Perisic et al., 1994). In the figure, Fc domains are not shown and each ScFv domain is represented with a different color (green, red, yellow or purple); green-red and yellow-purple represent the two different diabodies, while the connexin hemichannel is shown in blue. For additional structural details, see Xu et al. (2017).

**FIGURE 2:**
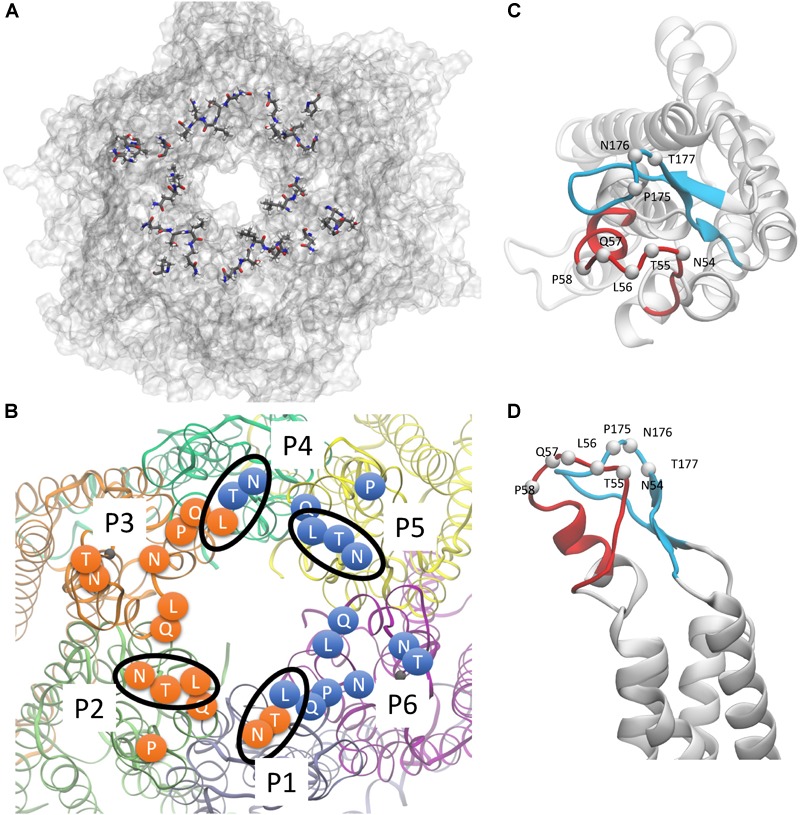
hCx26 hemichannel residues predicted to interact with abEC1.1. **(A,B)** Schematic representation of a hCx26 hemichannel viewed from the extracellular side. The most important hemichannel residues (percentage of interaction > 55%) interacting with abEC1.1 are highlighted. In panel **B**, different protomers (P1 to P6) of the hCx26 hemichannel are shown with different colors; labeled circles represent the position of the alpha carbon of residues shown in panel **A**; orange and blue residues represent the binding regions of the two scFv composing each diabody. Four N-T-L motifs are inscribed in an ellipse to indicate interaction of the corresponding hemichannel residues with either one (for P2 and P5) or both (for P1 and P4) binding diabodies. **(C,D)** Positions of all single protomer residues potentially involved in the interaction with the hemichannel (**C**, top view; **D**, side view); the EC1 loop is represented in red, the EC2 loop in light blue.

**FIGURE 3:**
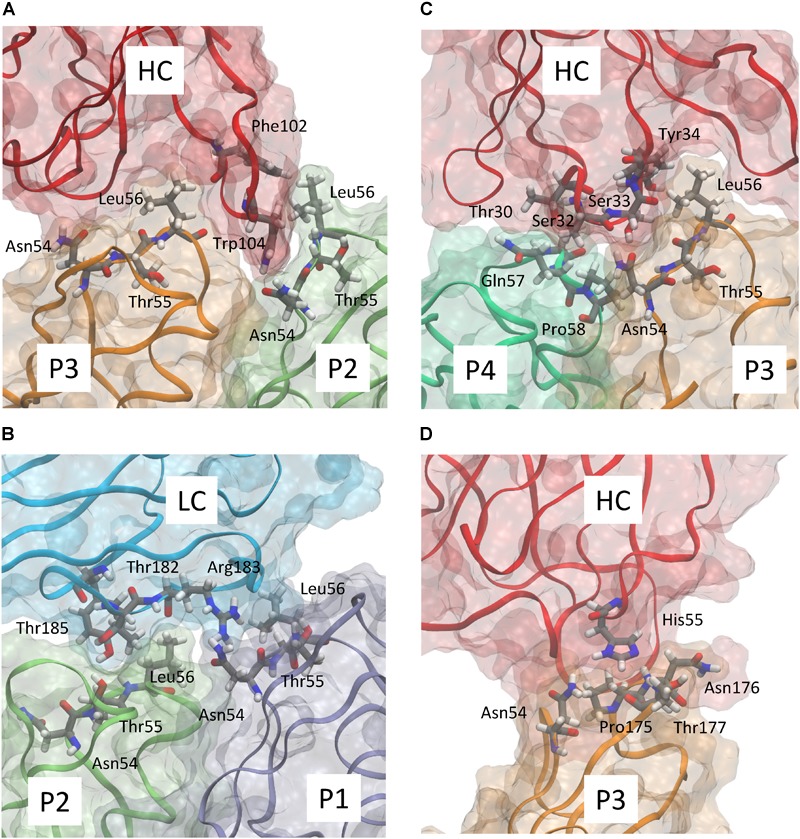
Detailed representation of the predicted interaction of abEC1.1 with the different protomers composing a hCx26 hemichannel. Panels **(A–D)** show four representative snapshots along the MD simulation highlighting the most important interactions; different protomers are represented according to the color code used in Figure 2B. The antibody heavy chain (HC) and light chain (LC) are represented in red and cyan, respectively. Each protein is represented according to its realistic volume occupation in space. Critical residues are shown with atomistic detail.

Starting from the equilibrated configuration of two abEC1.1 antibodies simultaneously docked to a hCx26 hemichannel (Xu et al., 2017) (Figure 1), we analyzed the last 10 ns of the MD simulation (see section “Materials and Methods”) and searched for antibody-connexin residue pairs that interacted stably. Interaction probability was measured as the fraction of the simulation time in which the distance between each pair of residues was less than an arbitrarily pre-assigned threshold (2 Å). Figure 2 highlights hemichannel residues involved in the interaction with abEC1.1 according to this scoring method. Based on this analysis, we concluded that abEC1.1 binds Asn54, Thr55, Leu56, Gln57, and Pro58 in EC1, as well as Pro175, Asn176 and Thr177 in EC2. Each antibody appears to interact directly with four different protomers (P) of the connexon: the first antibody interacts with P1, P2, P3, and P4; the second with P4, P5, P6, and P1, thus the pair of diametrically opposed protomers, P1 and P4, is able to interact with both binding antibodies at the same time. In other words, the stoichiometry of binding between connexins and antibodies is 6:2, with the two antibodies binding in a symmetric configuration relative to the axis of the channel pore. Details of the interactions are illustrated in Figure 3. Note that only EC1 residues Asn54, Thr55 and Leu56 were present in the bait peptide used to select abEC1.1 out of a phage display library with ∼10^11^ variability (Zhang et al., 2012). Sequence alignment of all human connexins showed that only hCx26, hCx30 and hCx32 display identical sub-sequences in the predicted binding region (Table 1). Therefore we reasoned that the antibody should inhibit also hCx30 and hCx32 hemichannels, whereas it might be less effective or ineffective when applied to hemichannels formed by other connexin isoforms.

**Table 1:** Sequence alignment of human connexin proteins in the regions where hCx26 is presumed to bind abEC1.1.

Gene Name	Protein name	EC1 amino acids 54–58	EC2 amino acids 175–176
*GJB2*	hCx26	NTLQP	PN
*GJB6*	hCx30	NTLQP	PN
*GJB1*	hCx32	NTLQP	PN
*GJB4*	hCx30.3	NTKQP	PH
*GJD3*	hCx31.9	NTLQP	PH
*GJA8*	hCx50	NTQQP	PN
*GJA3*	hCx46	NTQQP	PN
*GJA10*	hCx62	NTRQP	PN
*GJB5*	hCx31.1	NTRQP	PN
*GJB3*	hCx31	NTKQP	PN
*GJA9*	hCx59	NTEQP	PN
*GJC2*	hCx47	NTRQP	PH
*GJA1*	hCx43	NTQQP	PH
*GJA5*	hCx40	DTIQP	PH
*GJD4*	hCx40.1	NTLQP	TG
*GJC1*	hCx45	NTEQP	PH
*GJD2*	hCx36	NTLQP	IK
*GJG3*	hCx30.2	HTQQP	LG
*CJB7*	hCx25	NSRQP	PN
*GJA4*	hCx37	NTAQP	PY

*Residue Thr177 is part of the putative binding interface (see P3-EC2 in Table 2) but contributes only with its main chain, therefore it was not included in this table.*

### Experimental Validation of Model Predictions by Patch Clamp in HeLa DH Transfectants

To test these hypotheses, we performed a series of experiments in HeLa DH cells transiently transfected with the cDNAs encoding selected human connexins tagged with the Venus fluorescent protein at their C-terminus (see section “Materials and Methods”). The analysis was carried out on HeLa DH transfectants that generated reliable responses to a previously tested voltage clamp protocol (Xu et al., 2017) designed to elicit hemichannel currents upon depolarization followed by tail currents upon hyperpolarization.

**FIGURE 4:**
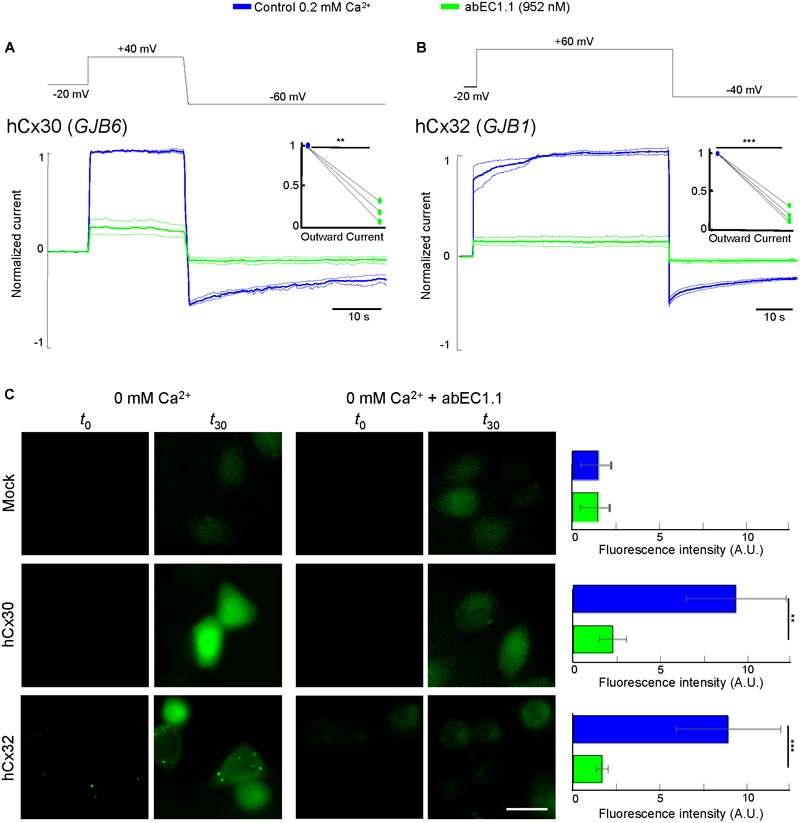
The abEC1.1 antibody inhibits hCx30 and hCx32 hemichannels in HeLa DH transfectants. **(A,B)** Whole cell currents elicited by voltage commands (top, black trace) recorded from HeLa DH cells transiently transfected with hCx30 **(A)** or hCx32 **(B)** and perfused with an extracellular solution containing 0.2 mM Ca^2+^ concentration. Shown are mean values (thick traces) ± s.e.m. (thin traces) for at least *n* = 3 cells in each data set. Total hemichannel currents were measured before (blue traces, control) and after (green traces) application of abEC1.1 by pressure at 952 nM for 15 min. Data were normalized to the mean value of the control response evoked by application of the depolarization step. Top insets show normalized outward currents measured immediately after the voltage commands to +40 mV **(A)** or +60 mV **(B)**. Asterisks indicate significance level between control condition and antibody application (^∗∗^*P* < 0.01; ^∗∗∗^*P* < 0.001, paired *t*-test). **(C)** Dye uptake assays (see section “Materials and Methods”). Cells were bathed in a divalent-free extracellular medium (ECM) or ECM + 952 nM abEC1.1. Shown are representative fluorescence images of the same field of view before (*t*_0_) and after 30 min of incubation with 2 mM Lucifer Yellow (*t*_30_). These experiments were performed twice for each type of HeLa DH transfectants (Mock, hCx30, hCx32). Histogram shows mean values ± s.e.m. of the difference between *t*_30_ and *t*_0_ fluorescence intensity values measured in 50 cells for each condition (A.U., arbitrary units; ^∗∗^*P* < 0.01, ^∗∗∗^*P* < 0.001, Mann–Whitney *U* test). Scale bar = 20 μM.

In cells expressing hCx30 (encoded by *GJB6*) and hCx32 (*GJB1*), whole cell current recordings showed a major reduction of the elicited currents in the presence of the antibody (952 nM; Figure 4A,B). Incubating hCx30 or hCx32 HeLa DH transfectants in Ca^2+^-free extracellular medium containing Lucifer Yellow (1 mM) promoted dye uptake, which was strongly reduced in cells that had been pre-incubated for 30 min with the antibody (952 nM; Figure 4C).

**FIGURE 5:**
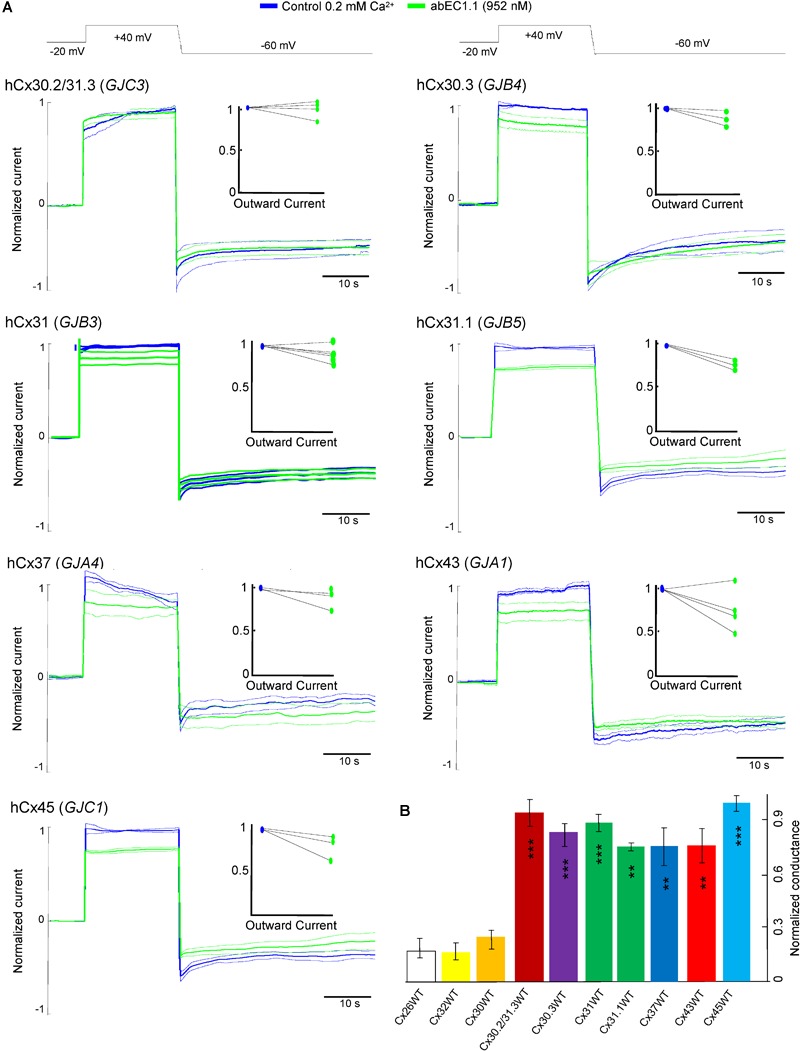
Specificity of the abEC1.1 antibody for different connexin hemichannels expressed in HeLa DH cells. **(A)** Black traces at the top represent voltage commands applied under patch clamp conditions in order to elicit whole cell currents. Shown are mean values (thick traces) ± s.e.m. (thin traces) of currents recorded in control conditions (blue traces) and after (green traces) abEC1.1 application by pressure at 952 nM concentration from a glass micropipette for 15 min. All data were normalized to the mean value of current elicited by the depolarization step to +40 mV. The effect of the antibody was tested on at least *n* = 3 cells, for each different connexin isoform. HeLa DH transfectants were superfused into an extracellular solution with low calcium concentration (0.2 mM). Top insets show normalized outward currents measured immediately after the voltage commands to +40 mV. Data revealed no statistical difference between control condition and antibody application. **(B)** Histograms (mean ± s.e.m.) represent membrane conductance computed from data shown in panel **(A)** and normalized to current values measured before antibody application. Asterisks indicate significance level (^∗∗^*P* < 0.01; ^∗∗∗^*P* < 0.001, ANOVA) for abEC1.1 effect on hemichannel currents measured in the indicated HeLa DH transfectants, taking hCx26 hemichannel currents in the presence of the antibody as reference.

At this concentration, the antibody was significantly less effective on all other connexin hemichannels of the pool we tested (Figure 5), namely hCx30.2/31.3 (*GJC3*), hCx30.3 (*GJB4*), hCx31(*GIB3*), hCx31.1 (*GJB5*), hCx37 (*GJA4*), hCx43 (*GJA1*), hCx45 (*GJC1*). Figure 5B provides a quantitative summary of these results in histogram form: residual hemichannel conductance following antibody application was equal to 16.8% for hCx26 (data from Xu et al., 2017), 16% for hCx32, 25% for hCx30, 91% for hCx30.2/31.3, 82% for hCx30.3, 87% for hCx31, 73% for hCx31.1, 73% for hCx37, 73% for hCx43 and 98% for hCx45.

**FIGURE 6:**
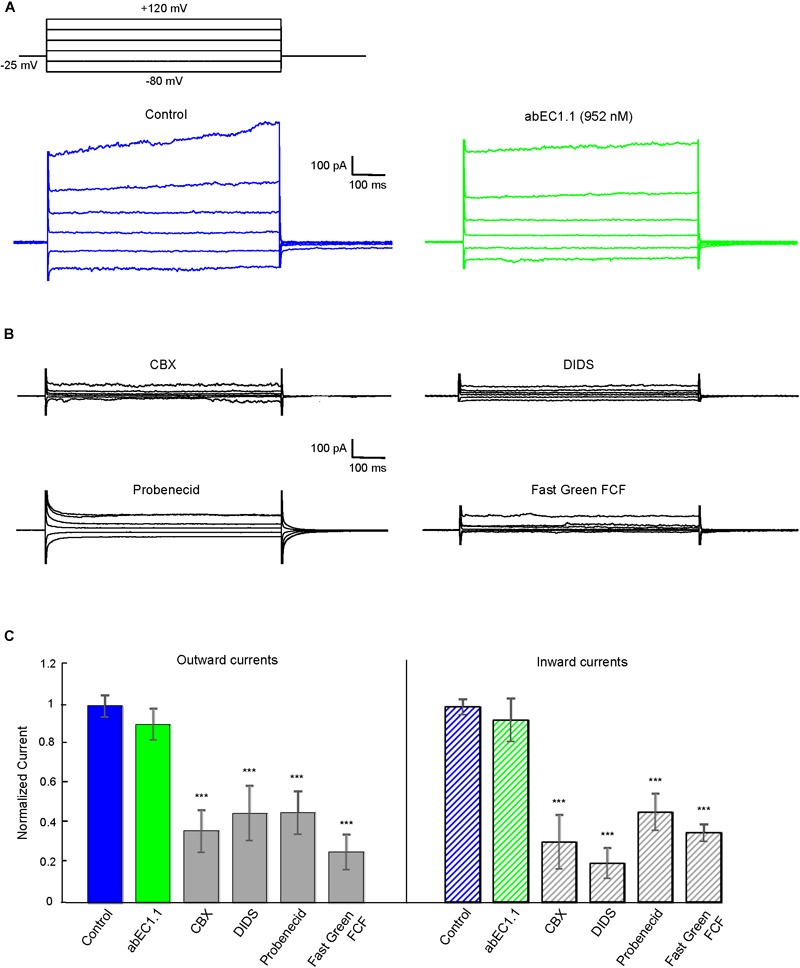
The abEC1.1 antibody does not affect human pannexin 1 (Panx1) hemichannel currents in HeLa-Panx1-YFP cells. Black traces at the top represent voltage protocols applied under patch clamp conditions to elicit whole cell currents in HeLa cells expressing Panx1. **(A)** Whole cell currents recorded in response to voltage steps from –80 to +120 mV before (control conditions) and after abEC1.1 antibody application at 952 nM for 15 min. **(B)** Inhibition of human Panx1 currents: shown are measurements of whole cell currents recorded in response to voltage protocol application after inhibitory compounds were delivered by pressure for 2 min (CBX, 50 μM) or 4 min (DIDS, Probenecid, Fast Green FCF, 100 μM). Results in panels **(A)** and **(B)** are representative of 4 to 8 experiments for each condition. **(C)** Histograms (mean ± s.e.m.) represent maximal outward and inward currents normalized to corresponding control values measured before antibody or pannexin inhibitor application. Asterisks indicate significance level relative to Control (^∗∗^*P* < 0.01; ^∗∗∗^*P* < 0.001, ANOVA).

Pannexin 1 is an alternative mediator of intercellular signaling (Barbe et al., 2006; Dando and Roper, 2009; Hanstein et al., 2013; Bravo et al., 2014; Beckel et al., 2015; Dong et al., 2016) with a pharmacology profile that overlaps partially that of connexin hemichannels (Dahl et al., 2013; Giaume et al., 2013). In HeLa-Panx1-YFP cells, which express no connexins (Yi et al., 2017), plasma membrane channels composed of pannexin 1 were not affected by abEC1.1 (applied at 952 nM concentration, Figure 6A), but were blocked by known non-specific pannexin inhibitors such as probenecid, carbenoxolone (CBX), 4,4-Diisothiocyanatostilbene-2,2-disulfonic acid (DIDS) and Fast Green FCF (Figure 6B,C). Altogether, these results indicate that abEC1.1 can discriminate between pannexin 1 channels and hemichannels composed of hCx26, hCx30 or hCx32.

### Assay of Antibody *in vitro* Stability by Size Exclusion Chromatography (SEC)

**FIGURE 7:**
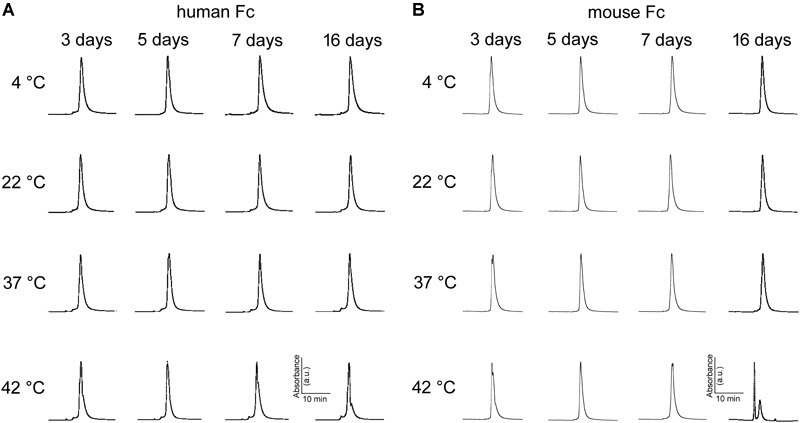
Peak-normalized SEC chromatographic profiles (absorbance vs. retention time) of antibodies maintained under different storage conditions. **(A)** abEC1.1; **(B)** abEC1.1m; see section “Materials and Methods” and Refs. therein.

Aggregation of monoclonal antibodies into high molecular weight species is an irreversible process that is notorious for its detrimental effects on antibody efficiency and safety (Beck et al., 2013). SEC is a widespread method for stability monitoring due to its short run times and quantitative reproducibility (Goyon et al., 2017). The peak-normalized chromatographic profiles (absorbance at 405 nm vs. retention time) in Figure 7A indicate abEC1.1 undergoes minimal or no aggregation for up to 16 days at temperatures comprised between 4°C and 37°C. Some abEC1.1 aggregates become noticeable for samples held at 42°C for 16 days. Figure 7B shows representative chromatographic profiles obtained from abEC1.1m, an antibody with the scFv domain of abEC1.1 fused in frame to a mouse Fc. A higher tendency to aggregate, signaled by the appearance of multiple peaks for abEC1.1m held at 42°C for 16 days, is evident at these rather extreme conditions. Altogether, these results confirm that abEC1.1 is a potential candidate protein biopharmaceutical.

## Discussion

### Anti-connexins Antibodies as Tools to Interfere With Hemichannel Function

A few monoclonal or polyclonal antibodies that recognize the extracellular domain of Cx43 have been generated (Meyer et al., 1992; Hofer and Dermietzel, 1998; Siller-Jackson et al., 2008; Baklaushev et al., 2009, 2011) and used in diverse contexts to address biological questions regarding gap junction assembly and hemichannel function [reviewed in Riquelme et al. (2013)], as well as for the potential treatment of cancer and inflammatory states (Jiang et al., 2018). Polyclonal “CELAb” antibodies raised in rabbits against a peptide corresponding to residues 42–53 in the EC1 region of hCx26 (VWGDEQADFVC), or another peptide corresponding to residues 172–184 in EC2 (AWPCPNTVDCFVSR), were used to detect Cx26 hemichannels in HeLa cells transfected with Cx26-eYFP, as well as to block dye uptake promoted by low Ca^2+^ conditions in both HeLa cells and CaCo-2/TC7 cells (Clair et al., 2008). The same antibodies detected connexin hemichannels also at the endolymphatic surface of the organ of Corti in mouse organotypic cochlear cultures (Anselmi et al., 2008; Majumder et al., 2010). However, to our knowledge, no translational follow-up has been developed using CELAb antibodies.

### Structural Determinants of abEC1.1-Hemichannel Interaction

abEC1.1 is the first connexin-binding human-derived monoclonal antibody that was selected from a vast phage display library (Zhang et al., 2012) using a bait peptide corresponding to residues 41-56 of EC1 in hCx26 (Qu et al., 2017). In the following, we will refer to hemichannel interactions with one of the two binding antibodies, keeping in mind that the pattern is symmetrical for the second antibody. Prior work allowed us to identify critical residues in the hemichannel-antibody binding interface, namely EC1 residues Asn54, Thr55 and Leu56, and antibody residues Tyr34, His55 and Trp104 (Xu et al., 2017). This work extends those results and suggests that two contiguous N-T-L motifs in P2-P3 accommodate the CDR3 of the antibody HC in a way that is reminiscent of hemichannel docking, owing in particular to the hydrophobic interactions of P2 and P3 Leu56 with Phe102 and Trp104 of the antibody. Likewise, P1 and P2 N-T-L motifs bind both the scaffold and, partially, the CDR2 loop of the antibody light chain, due to the formation of hydrogen bonds between antibody Arg183 and P1 Ans54, and to the interaction of antibody Tyr178 and Thr183 with Thr55 and Leu56 of P2. The conclusion based on this analysis is that the EC1 loop apex in all six protomers contributes to antibody binding (Figure 2, 3 and Tables 2, 3). Residues Thr30, Ser32, Ser33 and Tyr34 in the CDR1 of the antibody HC reside between the EC1 of P3 and P4 and occupy a more external position relative to the center of the channel, so that also Gln57 and Pro58 in EC1 and Pro175 in EC2 are involved in the antibody-connexon interaction. Finally, the CDR2 of the antibody HC interacts with P3 only through His55, which finds an equilibrium position between the apical part of EC1 (Asn54) and EC2 (Pro175, Asn176 and the main chain of Thr177). Note that, apart from the EC2 Asp179, all other EC1 and EC2 residues, which are critical for hemichannel docking and formation of intercellular channels, mediate also hemichannel binding to the antibody.

**Table 2:** Interaction probability of hCx26 residues with abEC1.1.

Hemichannel amino acid	Interaction probability
**P1-EC1**	
Asn54	82%
Thr55	56%
Leu56	85%
**P1-EC2**	
Pro175	29%
**P2-EC1**	
Cys53	10%
Asn54	92%
Thr55	82%
Leu56	100%
Gln57	78%
Pro58	31%
**P2-EC2**	
Pro173	2%
Pro175	68%
Asn176	21%
Thr177	4%
Val178	2%
**P3-EC1**	
Asn54	63%
Thr55	7%
Leu56	100%
Gln57	65%
Pro58	7%
**P3-EC2**	
Trp172	12%
Pro173	7%
Pro175	43%
Asn176	56%
Thr177	68%
**P4-EC1**	
Thr55	2%
Leu56	85%
Gln57	90%
Pro58	73%
**P4-EC2**	
Pro175	14%
Asn176	5%

*At blocking concentrations, two antibodies bind simultaneously a single hCx26 hemichannel (Xu et al., 2017). Different hCx26 protomers (P1 to P6) contribute to the binding interface with different residues. One of the two antibodies binds protomers P1-P2-P3-P4; the second binds protomers P4-P5-P6-P1 with an identical interaction pattern. The interaction probability was computed as the time fraction the listed hCx26 residues were in close contact with the antibody during the MD simulation.*

**Table 3:** Interaction probability of abEC1.1 residues with the extracellular domain of the hCx26 hemichannel.

Antibody amino acid	Interaction probability	Antibody amino acid	Interaction probability
**HC Constant Region**		**LC Constant Region**	
Ser27	19%	Tyr178	80%
Asn76	14%	Thr182	98%
Ser77	5%	Arg183	93%
Asn79	2%	Ala184	58%
		Thr185	85%
**HC-CDR1**		Gly186	68%
Gly28	29%	Ile187	43%
Phe29	53%	Pro188	63%
Thr30	100%	Asp189	85%
Ser32	70%		
Ser33	98%	**LC-CDR1**	
Tyr34	98%	Ser160	5%
**HC-CDR2**		**LC-CDR2**	
His55	73%	Gly179	2%
		Ser181	37%
**HC-CDR3**			
Phe102	78%		
Ser103	90%		
Trp104	100%		
Arg105	17%		

*The interaction probability was computed as the time fraction the listed abEC1.1 residues were in close contact with extracellular domain of the hCx26 hemichannel during the MD simulation. HC, heavy chain; LC, light chain; CDR, complementarity determining region.*

Together with sterical hindrance arguments, this analysis provides a mechanistic cue for the observed strong reduction of electrical conductance and dye uptake for hCx26 (Xu et al., 2017), hCx30 and hCx32 hemichannels (this work), which have 100% identical sequences in the predicted binding region (in hCx30 the amino acid corresponding to Thr177 is a Leu, but the interaction with the antibody is only mediated by the oxygen and nitrogen of the main chain).

Summarizing, the pattern of interaction is remarkably rich and the repeated N-T-L sequence binds different regions of the antibody. A single amino acid difference in a limited group of 8 protomer residues is sufficient to reduce significantly the antibody effects on all other types of hemichannels tested. Residues belonging to the hemichannel and the antibody which contribute most to the interaction are shown in Tables 2 and 3, respectively.

### Antibody Selection Based on Phage Display Library Screening: Potential and Caveats

The atomistic model presented here, validated by patch clamp measurements, imply that it is possible to identify hemichannel blocking antibodies which are specific at least for different connexin subfamilies. However, our results also imply that screening antibody libraries against a simple bait peptide does not guarantee that the selected antibodies recognize the hemichannel surface exposed to the solvent. Indeed, it is well known that successful isolation of novel antibodies by phage display is critically limited by the presentation of a correctly folded antigen. The discovery of new and more selective antibodies capable of interfering with connexin hemichannels will undoubtedly benefit from improved methods targeting membrane proteins in their native conformation and optimized for whole cell biopanning (Jones et al., 2016, 2018).

## Concluding Remarks

Given that abEC1.1 is stable *in vitro*, we consider the results presented here as potentially relevant for disorders caused by augmented hemichannel activity of hCx26 (Stong et al., 2006; Gerido et al., 2007; Lee et al., 2009; Mhaske et al., 2013; Sanchez et al., 2014; Garcia et al., 2015), hCx30 (Essenfelder et al., 2004) and hCx32 (Abrams et al., 2002; Liang et al., 2005), which include *Keratitis-ichthyosis-deafness syndrome* (OMIM 148210), *Hystrix-like ichthyosis with deafness* (OMIM 602540), *Ectodermal displasia 2, Clouston type* (OMIM 129500), and *Charcot-Marie-Tooth disease, X-linked dominant, 1* (OMIM 302800). *In vivo* experiments with relevant animal models will be key to test the therapeutic potential of this antibody.

## Author Contributions

GY, FM, FZ, and RAL designed the studies and provided resources to conduct the studies. GZ, DB, AC, and CN performed the electrophysiology experiments. GZ and CN performed the dye uptake experiments. FC subcloned connexin plasmids in the pcDNA3.1 transfection vector and performed quality controls on plasmids. LX subcloned connexin plasmids in the pcDNA3.1 transfection vector and produced antibodies. YK produced antibodies and performed size exclusion chromatography experiments. DB and FZ performed the molecular dynamics simulations. AS, FM, GP, FZ, GY, and RAL supervised the work of junior colleagues. GP provided critical feedback and helped to editing and revising the manuscript. FM and FZ wrote the manuscript.

## Disclaimer

Aspects of this work are encompassed by patent application PCT/CN2019/088689 application date May 28, 2019.

## Conflict of Interest Statement

RAL is a founder of Zebra Biologics Inc. GY is a partner of Zebra Biologics Inc. The remaining authors declare that the research was conducted in the absence of any commercial or financial relationships that could be construed as a potential conflict of interest.

## Footnotes

1 As listed at https://www.ncbi.nlm.nih.gov/nuccore.

## Abbreviations

3D: three-dimensional
[Ca^2+^]: calcium ion concentration
Ca^2+^: calcium ion
CBX: carbenoxolone, 3β-Hydroxy-11-oxoolean-12-en-30-oic acid 3-hemisuccinat
CDR: complementarity determining regions
CTD: C-terminal domain
Cx: connexin
DIC: differential interference contrast
DIDS: 4,4-Diisothiocyanatostilbene-2,2-disulfonic acid
DMEM: Dulbecco’s modified Eagle’s medium
EC1,2: extracellular loop 1, 2
ECS_NaCl_: sodium chloride-based extracellular solution
FBS: fetal bovine serum
Fc: fragment constant
HC: heavy chain
ICL: intracellular loop
ICS_KAsp_: potassium aspartate-based intracellular solution
IgG1: immunoglobulin G1
LC: light chain
MD: molecular dynamics
NTH: N-terminal helix
OD: optical density
ORF: open reading frame
P1-6: protomers 1 to 6 of a homomeric hCx26 hemichannel
PBS: phosphate-buffered saline
ScFv: single chain fragment variable
SEC: size exclusion chromatography
TEA-Cl: 4 tetraethyl ammonium chloride
TM1-4: transmembrane helix 1 to 4
Wt: wild type
YFP: yellow fluorescent protein
